# Investing in Onchocerciasis Control: Financial Management of the African Programme for Onchocerciasis Control (APOC)

**DOI:** 10.1371/journal.pntd.0003508

**Published:** 2015-05-14

**Authors:** Donald A. P. Bundy, Bilkiss Dhomun, Xavier Daney, Linda B. Schultz, Andy Tembon

**Affiliations:** 1 World Bank Group, Washington, D.C., United States of America; 2 World Health Organization, Geneva, Switzerland; Liverpool School of Tropical Medicine, UNITED KINGDOM

## Introduction

The African Programme for Onchocerciasis Control (APOC) has been described as one of the most successful public-private partnerships for health in Africa [1]. The Programme helps the governments of 20 onchocerciasis-endemic countries to develop sustainable delivery of ivermectin treatment in order to control and eliminate river blindness [2]. APOC is one of a very few programs that the WHO implements directly, in this case through the Regional Office for Africa (AFRO) and the APOC Secretariat established in Ouagadougou, Burkina Faso. As the Executing Agency of APOC, WHO is responsible for the development of procedures for implementing community-directed treatment with ivermectin (CDTi), approving funding to countries, maintaining surveillance, and ensuring monitoring and evaluation [3]. The Executing Agency supports governments directly in this role, aided by a group of 15 Non-Governmental Development Organizations (NGDOs), operating nationally, regionally, and globally [4]. The 15 NGDOs include Charitable Society for Social Welfare, Christoffel-Blindenmission (CBM), Helen Keller International (HKI), Light the World, Mectizan Donation Program (MDP), Mission to Save the Helpless (MITOSATH), Organisation pour la Prévention de la Cécité (OPC), Sightsavers International, United Front Against Riverblindness (UFAR), Interchurch Medical Assistance (IMA) World Health, Lions Clubs International Foundation, Malaria Consortium, Schistosomiasis Control Initiative, The Carter Center, and The United States Fund for UNICEF. The funding for national onchocerciasis control comes from the countries and civil society, with additional funding from APOC through contributions from more than 30 development partners to a Trust Fund that the World Bank manages in its role as Fiscal Agent.

Scheduled to close in 2015, the main governing body of APOC decided to set up a new programme for a ten-year period from 2016, actively seeking to eliminate river blindness from most of Africa by 2025 and, by strengthening the same health system that successfully delivered treatment for river blindness, seeking to also eliminate lymphatic filariasis and co-implement treatments for the five other major preventable neglected tropical diseases (NTDs) that can be addressed through mass drug administration. Plans are underway to transform APOC beyond 2015 and transition from a single-disease entity to an expanded regional NTD initiative [5].

This article is one of a series developed by the partnership of agencies, institutions, and individuals that make up APOC. This article is written from the perspective of the Fiscal Agent (the APOC Team at the World Bank) and should be read in conjunction with the corresponding articles in this series that examine the depth and diversity of the river blindness partnership, which includes the Executing Agency (World Health Organization) [3], civil society [4], and the Mectizan Donation Program [6].

## The Origins of Financial Management of Onchocerciasis Control in Africa

The origins of financial support for onchocerciasis control can be traced back to those of World Bank’s support for health in general. The International Bank for Reconstruction and Development (IBRD)—the first institution of what was later to become the World Bank Group (WBG)—was founded in 1944 with the primary aim of supporting post—World War II reconstruction and economic development. In the 1960s, the World Bank extended its remit to specific support for low-income countries by the provision of concessional credits and grants through the International Development Association (IDA).

The main focus of the World Bank’s support then was on infrastructure projects. The earliest grants relevant to health and human development were minor investments in water and sanitation projects in the 1960s and support for family planning in the 1970s [7]. In January 1972, the World Bank published a report, Possible Bank Actions on Malnutrition Problems, which led to the establishment of a nutrition department within the World Bank, and in 1975 the World Bank released its first *Health Policy* paper creating the combined health, nutrition, and population structure that exists today as the HNP Network. The World Bank’s health portfolio has steadily increased since those early beginnings: from an average of US$25 million per year in the 1970s, to US$1.4 billion per year in the 1990s, and rising to US$1.7 billion in the 2000s [8].

It was against this background that support for the Onchocerciasis Control Programme for West Africa (OCP) was launched in 1974, arguably the first health-focused project that the World Bank supported [9]. Political support for this step has been attributed to a visit made by the then—World Bank President Robert McNamara to Burkina Faso, where he was shocked to see numbers of blind adults being led with a stick by their children, an iconic image of river blindness that then, as now, did much to raise public awareness of the disease. Bronze statues depicting this image are displayed in multiple institutions around the world that have made an important contribution to the control of river blindness.

## The Onchocerciasis Control Programme for West Africa (OCP) and the Evolution of the African Programme for Onchocerciasis Control (APOC)

**Fig 1:**
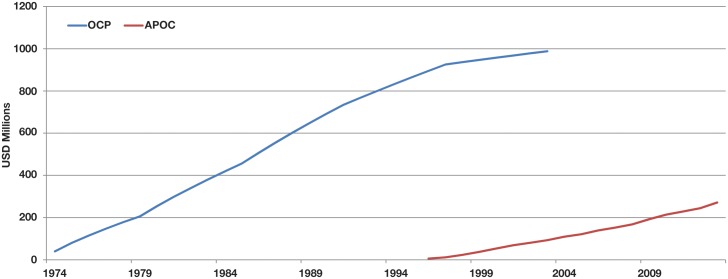
APOC and OCP total cumulative donations (1974–2013).

In 1974, OCP used vector control with larvicides to control river blindness in 11 countries in West Africa. The 11 countries included Benin, Burkina Faso, Cote d'Ivoire, Ghana, Guinea, Guinea Bissau, Mali, Niger, Senegal, Sierra Leone, and Togo. The Executing Agency was WHO, with the World Bank working with other development agencies as Fiscal Agent, essentially under the same division of labor as exists today with APOC. OCP was very successful in controlling the disease, freeing 18 million children from the risk of blindness and reclaiming some 25 million hectares of abandoned arable land—enough to feed about 17 million people [10]. But expansion of OCP to the other 20 onchocerciasis-endemic countries in Africa was not feasible, as costs for the OCP countries alone had spiraled to nearly a billion US dollars (Fig 1) and the riverine vector control methods for the savannah onchocerciasis in the OCP area were not applicable to the forest onchocerciasis found in other regions. These countries included Angola, Burundi, Cameroon, Central African Republic, Chad, Congo, Democratic Republic of the Congo, Equatorial Guinea, Ethiopia, Gabon, Kenya, Liberia, Malawi, Mozambique, Nigeria, Rwanda, Sudan, Tanzania, Uganda, and South Sudan (which became the 20th participating country as of 2011).

In 1987, a new treatment-based strategy emerged, based on the recognition that the drug ivermectin had efficacy against onchocerciasis and the extraordinary commitment by Merck & Co., Inc., (MSD outside the US and Canada) to donate ivermectin (Mectizan), “as much as is necessary for as long as is necessary” [11]. Based on the effectiveness and lower cost of the treatment approach, APOC was launched in 1995, and in 2002 was extended into a second phase, APOC II, through 2015. For 40 years, the World Bank has played a key role as the fiscal agent for OCP and then APOC, bringing to bear its comparative advantage in fiscal governance by managing the APOC Trust Fund, which channels the contributions of more than 30 donors, including governments, foundations, philanthropists, and the private sector, to APOC.

## The APOC Trust Fund

The key financial instrument for managing APOC finances is its Trust Fund, a mechanism through which several donors can co-finance international development projects. The first World Bank trust fund was established in 1960 to finance the Indus Basin Project in Pakistan, providing US$90 million over 15 years. Trust fund management today plays a major role in World Bank support for development, with fund flows totaling US$29.1 billion in 2011 [12]. Some of the largest of these funds support health investments, such as the US$2.45 billion Global Fund to Fight Aids, Tuberculosis and Malaria (GFATM); the US$211 million International Finance Facility for Immunization (IFFIm); and the US$137 million Pilot Advance Market Commitment for Vaccines against Pneumococcal Diseases (AMC) [13]. The trust fund established in 1974 to help control onchocerciasis, covering both the OCP and the APOC period, has accumulated receipts to date of over US$1.2 billion (Fig 1).

For about half the trust funds, those that are Bank-Executed or are Recipient-Executed with bank oversight, the World Bank not only manages the fund but also implements or supervises the activities that are financed by the fund. In these types of funds the World Bank is responsible for both financial and programmatic functions, ultimately exercising oversight over the operational use of the funds [14].

**Fig 2:**
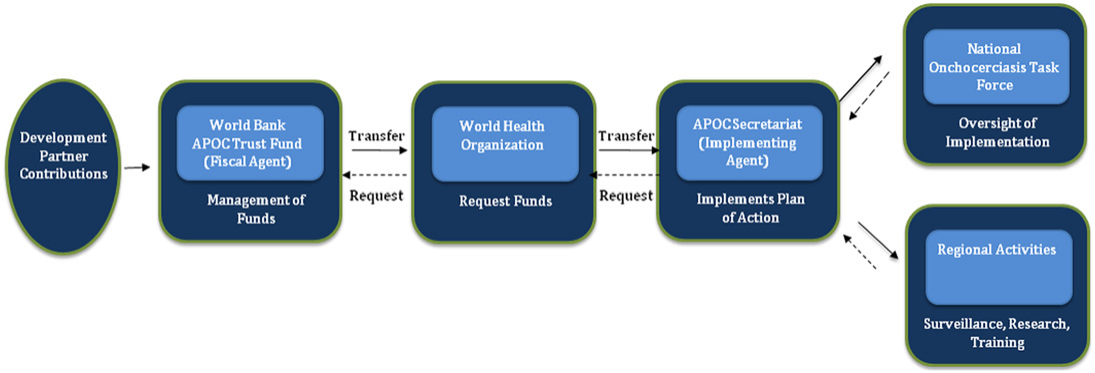
The flow of funds from donors to national and regional APOC activities.

For the remaining trust funds, the World Bank serves as a trustee with responsibility for the fiduciary oversight of the funds. These are termed Financial Intermediary Funds (FIF), and the APOC Trust Fund and the OCP Trust Fund before it are of this type. For all FIFs, the World Bank provides a set of agreed financial services that involve receiving, holding, and investing contributed funds, and then transferring those funds when requested by the Executing Agency. Under some FIFs, the Bank also provides other financial services, such as bond issuance, hedging intermediation, and monetization (e.g., of carbon credits). FIF Trusteeship does involve fiduciary oversight, but not the actual use of the funds, which is the responsibility of the implementing agency [14]. Fig 2 illustrates the flow of funds in the case of the APOC and OCP Trust Funds: the Fiscal Agent (World Bank) receives, holds, and invests funds from development partners and, on request, transfers these funds to the Executing Agency (WHO), from which they flow to the implementing levels (the participating countries and the APOC Secretariat).

## APOC Governance

APOC has strong representative governance. The ultimate decision-maker is the Joint Action Forum (JAF), which is comprised of the 20 governments of the participating countries represented at the level of Ministers of Health, as well as representatives of more than 30 contributing development partners, co-sponsoring agencies, and 15 NGDOs (that also formed into the NGDO Coordination Group for Onchocerciasis Control) that help implement and support the program [15]. The annual meeting of JAF makes the main policy decisions, in particular, the approval of plans of action and budgets, in accordance with legal proceedings overseen by the WHO Secretariat. There have been 19 meetings of the JAF up to the end of 2013.

To provide continuous oversight of the implementation of these policy decisions, a Committee of Sponsoring Agencies (CSA) meets four times per year to review reports of various organs and participants, acting on behalf of the JAF when required. The CSA is currently chaired by WHO and includes representatives of WHO (the Executing Agency), World Bank (the Fiscal Agent, also representing the contributing development partners), the African Development Bank, the NGDO coordination group, the Mectizan Donation Program and Merck & Co., Inc., with the assistance of the WHO’s Legal Office. There have been 145 meetings of the CSA through July 2014. This institutional framework is completed by a Technical Consultative Committee (TCC) meeting twice a year at APOC headquarters and providing technical advice to the Programme’s management.

**Fig 3:**
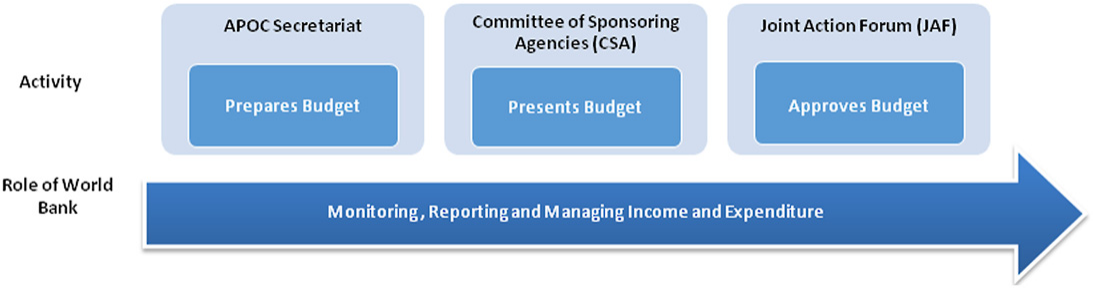
Process of budget preparation, approval, and monitoring.

The APOC Secretariat (as Executing Agency) is responsible for the implementation of activities and for initiating the budget process (Fig 3). The APOC Secretariat takes the lead in preparing a multi-year Plan of Action for APOC and uses this to develop an indicative budget to implement the multi-year plan (the APOC Plan of Action and Budget, PAB). To aid financial planning, a detailed budget is prepared on a two-year basis and provided to the CSA for the next step in the budget process. The biennial PAB is subject to scrutiny by the CSA, and a final version, agreed between the CSA and the APOC Secretariat, is presented to the JAF for formal approval. All financial transfers within APOC systems are made on the basis of this approved PAB.

## The Role of the Fiscal Agent

The primary role of the World Bank is as the fiscal agent for APOC. The World Bank is represented on different organs of APOC governance, but does not define or approve WHO requests for funding. In particular, the World Bank does not prepare or approve the PAB (Fig 3), although it is part of the oversight and scrutiny of the process. There is therefore a clear separation (within the APOC organizational framework) between the fiscal and implementing roles.

**Fig 4:**
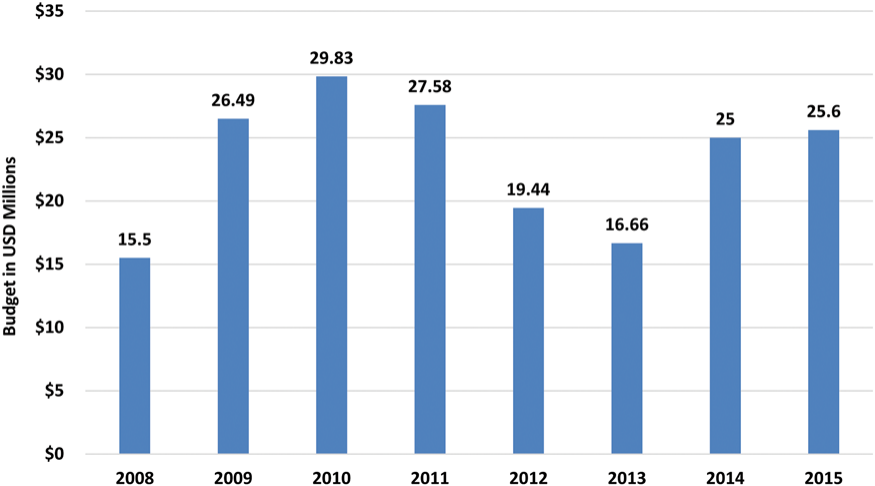
Current APOC strategic plan and budget, 2008–2015.

The PAB prepared by the APOC Secretariat and approved by JAF is the basis of all financial actions by the Fiscal Agent. The current PAB covers the period 2008–2015 (Fig 4) and indicates the anticipated expenditure to implement APOC activities over that period. A more detailed biennial budget, again prepared by the APOC Secretariat and approved by the JAF, guides precise expenditures over a two-year cycle. Based on requests from the WHO within the approved budget, and in accordance with the approved PAB, the World Bank transfers funds from the APOC Trust Fund to the WHO so that the APOC Secretariat may in turn support implementation at the regional and country levels.

The key instrument, which sets out the relationship between the fiduciary role of the World Bank and the implementing role of WHO, is the Memorandum for APOC. The Memorandum stipulates the role of the World Bank as providing fiduciary (legal) oversight of the APOC Trust Fund and that of WHO as ensuring the implementation of activities approved by APOC governance. This instrument is the basis of the agreement between contributing parties and the participating countries. In practice, decisions of a legal nature are made upon the advice of the World Bank and WHO legal teams. The World Bank ensures that the pooled trust fund has an adequate amount to cover the scheduled withdrawal requests from WHO and monitors and reports to the APOC Governing Bodies on the flow of funds for the program, helping ensure that these flows respond to the PAB approved by the JAF.

The World Bank also plays a role, in collaboration with the other APOC partners, in resource mobilization. Once the PAB has been approved by the JAF, the World Bank leads a joint effort by the APOC partnership to identify income and pledges of income for the duration of the PAB. The World Bank aims to respond effectively to the needs of existing development partners and to work with them and new partners to increase revenues, in particular when the JAF determines a need for additional support. All new development partners contributing to the Trust Fund are subject to World Bank requirements for due diligence and sign the standard Memorandum for APOC.

Throughout the duration of the OCP and APOC Trust Funds, both programs have enjoyed the enthusiastic support of multiple development partners that have been able to match the budgetary requests from the JAF. Until the decision to transform APOC was taken, funding and pledges of funding fully covered the PAB through the scheduled sunset of APOC in 2015.

The World Bank has a specific responsibility to the contributing development partners for the fiduciary oversight of the Trust Fund. In this context, the External Auditor of WHO conducts an annual independent audit of APOC’s financial statements, reporting to contributors at the JAF. The Fiscal Agent reports regularly to the contributing development partners on the progress of the CSA meetings and annually presents to the JAF an analysis of the fiscal health of the Trust Fund and its status in respect to the PAB.

**Fig 5:**
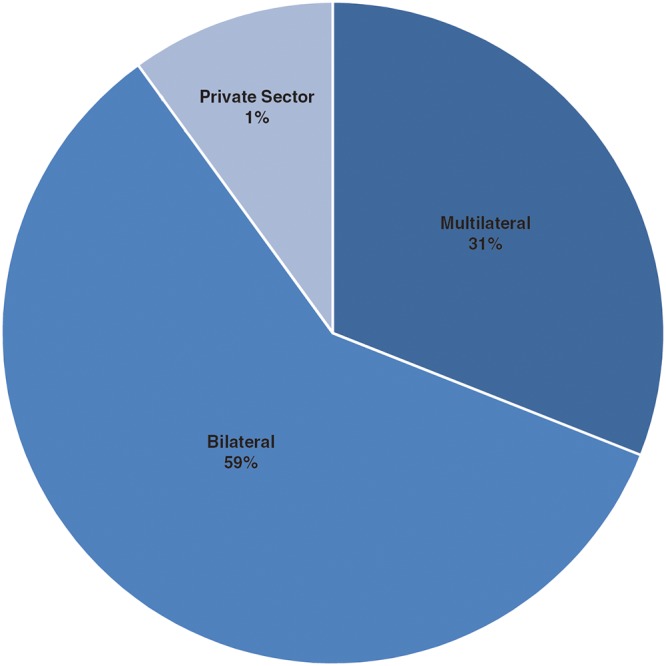
Contributions to the trust fund based on donor characteristics.

In addition to the management role, the World Bank is itself a contributor to the Trust Fund through its Global Partnership Programs (GPP) facility (Fig 5). Apart from this direct support to APOC, the GPP has also provided substantial indirect support to the work of WHO and the APOC Secretariat through its contributions to the Programme for Research and Training for Tropical Diseases (TDR), founded in 1975 by the World Health Organization with the United Nations Children's Fund (UNICEF), the United Nations Development Program (UNDP), and the World Bank. Research supported by TDR has contributed to the development of onchocerciasis control strategies, especially the work in collaboration with Merck and the Mectizan Donation Program, which was pivotal in the development of the use of ivermectin for onchocerciasis and in the development of the current CDTi mechanism, respectively.

## Financing of the APOC Trust Fund

**Fig 6:**
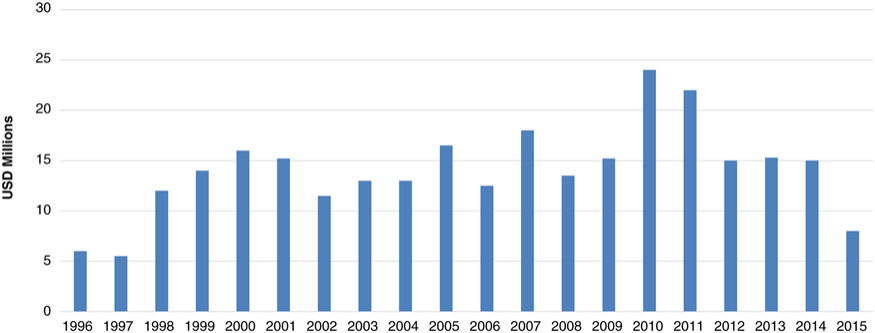
Total receipts: APOC I and II (1996–2015). Values from 1996–2011 have been deflated using the gross domestic product (GDP) deflator. 2012–2015 values represent the nominal amount.

The APOC Trust Fund is supported entirely through voluntary contributions from development partners that have been formally admitted as contributors to the Program. The Trust Fund was established in 1995 and is expected to have managed approximately US$258 million by 2015. Fig 6 shows the annual receipts since 1996, averaging US$13.0 million per annum. Together, the OCP and APOC trust funds have managed more than US$1.2 billion in support of river blindness control over the last 30 years (Fig 1).

**Fig 7:**
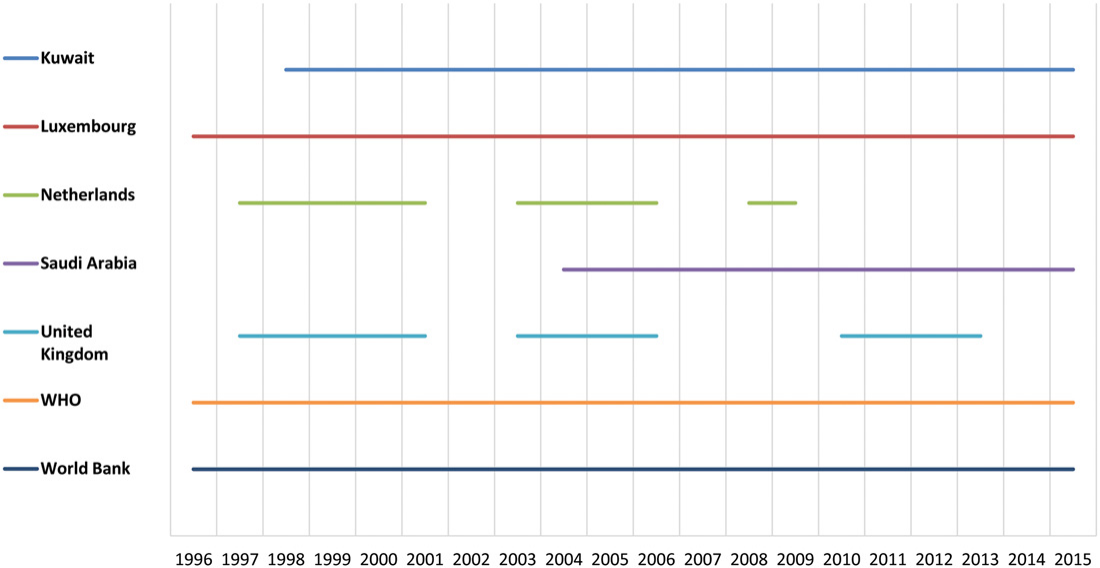
Annual contributions by selected donors during the period 1996–2015.

Effective control requires that treatment programs are sustained over a period of many years, and hence consistency, loyalty, and timeliness of contributions have been important factors in the success of APOC. While the scale of these large donations has been very important to the success of the program, there are other factors that have made some relatively smaller donations critical to the rollout of APOC. Several development partners, including quite a few of those making relatively small contributions, have individually provided the type of reliable and predictable support that has made long-term intervention possible (Fig 7). For instance, the Kuwait Fund has been a consistent donor since the beginning of the OCP program and has continued throughout APOC. Taken together, these relatively smaller contributions total an important 20% of the overall budget.

**Fig 8:**
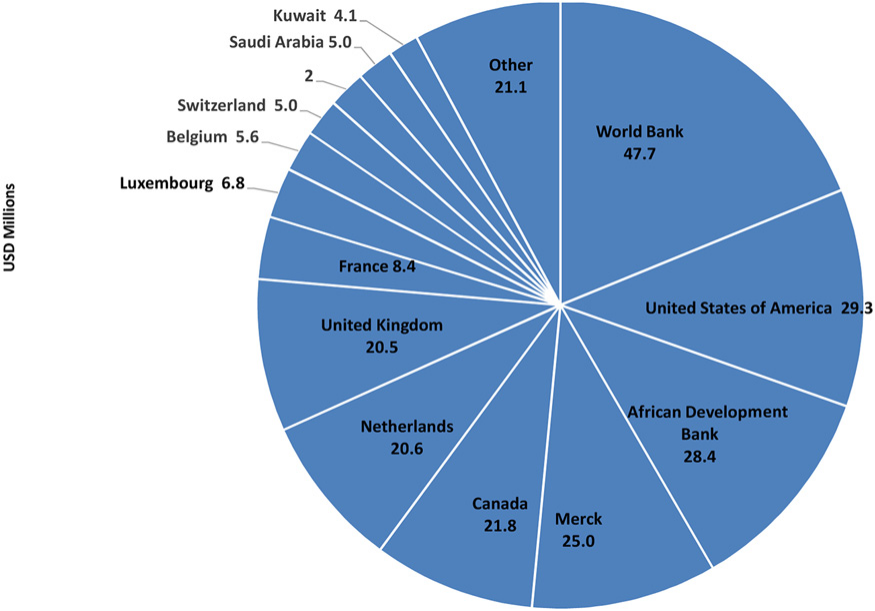
Contributions by APOC development partners to the Africa Regional Trust Fund for River Blindness, 1995–mid-2013. Total funds mobilized from 1995 to present: $225 million. In nominal US dollars. Other: Australia, BADEA, Caritasverband, Champalimaud Foundation, END Fund, Finland, Germany, Gulbenkian Foundation, Ireland, Japan, Kitosath Institute, MITOSATH, Norway, OFID, Poland, Portugal, Sabin Vaccine Institute, Sightsavers, Slovenia, UNDP, and WHO.

A key contributor to the success of APOC is the diversity and depth of its public-private partnership, which includes multilateral and bilateral agencies, development institutions, international organizations, private foundations, civil society and the private sector. Bilateral agencies are the largest single source of contributions (Fig 5). To date, the Governments of 20 countries have contributed to APOC Trust Fund, which contradicts the anecdotal view that there has been limited political interest in contributing to the control of NTDs (Fig 8).

Overall, 34 development partners have contributed to the APOC Trust Fund. Seven of these partners—World Bank, the United States, African Development Bank, Merck, Canada, the Netherlands, and the United Kingdom—have together contributed 80% of the total funds received by the Trust Fund. Some of the reasons why these partners have supported APOC so strongly are suggested by statements made by representatives of these organizations (Box 1).

**Box 1. Why Do the “Big Seven” Donors Support APOC?**

**World Bank**

“The APOC program is one of the most successful public-private partnerships in health in Africa.” Shanta Devarajan, Chief Economist, The World Bank Africa Region.

**United States (USAID)**

“The United States today is joining more than 40 non-governmental organizations, academic institutions, global health and civil society organizations to hail historic progress, celebrate champions, and underscore continuing challenges in the global fight against diseases affecting the world’s poorest and most marginal populations.” US Agency for International Development (USAID) Press Office.

**African Development Bank (ADB)**

“The challenge is the need to strengthen the successful public-private partnership realized by APOC which could only maintain all the gain obtained during more than 30 years for controlling Onchocerciasis. Among the major and sustainable gain is the reduction of inequalities and disparities that will lead to poverty alleviation, economic development, productivity growth, and social returns.” Office of the Principal Health Analyst, Human Development Department, Health Division, African Development Bank Group.

**Merck**

"We are pleased to augment our unwavering commitment to donate MECTIZAN (ivermectin) to all who need it, wherever needed, until river blindness is eliminated, by providing financial support to APOC. Only through partnership—working collaboratively and harnessing our complementary skills and resources—can we effectively address the most pressing global public health challenges." Kenneth C. Frazier, Chairman, President and Chief Executive Officer, Merck.

**The Netherlands**

“APOC has been very instrumental in bringing health, and in particular health systems, on the agenda of the Bank. The Programme made it clear that health is not just a ‘debit’ but a true asset, worth investing in. Large areas of land that were abandoned were used again for agriculture once river blindness had successfully been beaten. It proved that a bank could and should play a natural role in health Programs at global and country level. Not just as a trustworthy accountholder, but as a partner.” Office of Head of the Health and Aid Division, Social Development Department, Ministry of Foreign Affairs, Government of the Netherlands.

**United Kingdom (DFID)**

“It is a tragedy that the lives of millions of the world’s poorest people are still being destroyed by these ancient and avoidable tropical diseases (NTDs) when we have the means to tackle them… The world is increasingly coming together to build on the long-standing commitment of the pharmaceutical industry to rid the world of these terrible diseases which disable, blind, and kill millions every year… British support will take the neglected out of neglected tropical diseases and will not just save lives—but transform lives.” Stephen O’Brien, International Development Minister.

While the majority of support to the APOC Trust Fund is from public funds, private sector contributions make up a quarter of the total. Notably, this includes direct financial contributions from Merck (US$25 million, 2008–2015), which are additional to the value of the treatment donated through the Mectizan Donation Program. The annual value of treatment contributed by Merck to APOC, calculated at $1.50 per tablet in 2001, is estimated to be $146.6 million [16]. Since the treatment is donated, delivery of ivermectin is exceptional value for money.

It is worth noting that both the Fiscal Agent and the Executing Agency are also contributors. The World Bank and WHO make no charges on the Trust Fund; all of their services are supported directly by the World Bank budget. The World Bank does not levy any overhead on the income to the Trust Fund, and therefore 100% of the contributions enter the fund. In fact, the held funds typically augment, pending transfer to WHO, as the World Bank Treasury invests the funds prudently, when investment is allowed by the donor, as part of its overall management. To date, APOC funds have accrued an additional US$9.6 million in income from this source.

## The Contribution of the APOC Trust Fund to National Control Programs

The primary focus of support for national health systems in a given country is the national budget. In the case of onchocerciasis, three main channels through which development assistance is provided to support national health efforts to achieve onchocerciasis control can be identified: first, through bilateral aid to government health budgets (e.g., from bilateral, intergovernmental, and multilateral development agencies); second, through the regional trust fund created by APOC, receiving funding from multiple sources within and beyond the region; and third, indirect support from technical and civil society organizations (NGDOs), such as members of the NGDO Coordinating Group and technical assistance organizations, which themselves are supported by bilateral and multilateral development partners, private sector organizations, and their own fundraising efforts.

**Fig 9:**
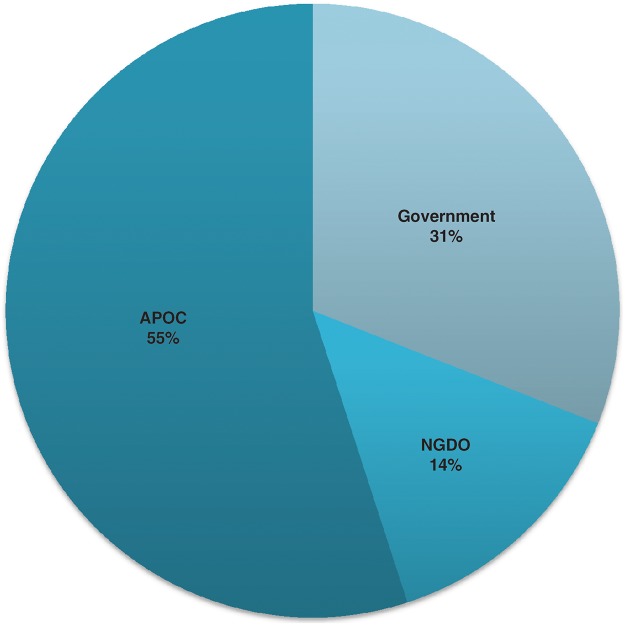
Sources of investment in national onchocerciasis control programs.

Fig 9 explores the relative contribution of these different channels to national onchocerciasis control efforts. Obtaining accurate data on budgetary commitments is difficult in general, and particularly when attempting to separate out support for a single intervention delivered through a health system, such as delivering treatment for onchocerciasis. Work supported by USAID is contributing to this area through the Tool for Integrated Planning and Costing (TIPAC), and WHO/AFRO has recently undertaken a regional costing exercise in 32 countries, based on national plans. At the request of the participating countries on the JAF of APOC, the African Development Bank and the World Bank are providing technical assistance to the APOC Secretariat to help countries estimate their current investments in onchocerciasis control. The countries have asked for this assistance to help them track their specific investment in onchocerciasis and in NTD control generally. According to the preliminary estimates of investment in onchocerciasis control presented in Fig 9, government investments, including bilateral budget support, currently cover about 31% of the total; 14% is provided by indirect investments, principally via civil society; and 55% is through the APOC channels.

**Fig 10:**
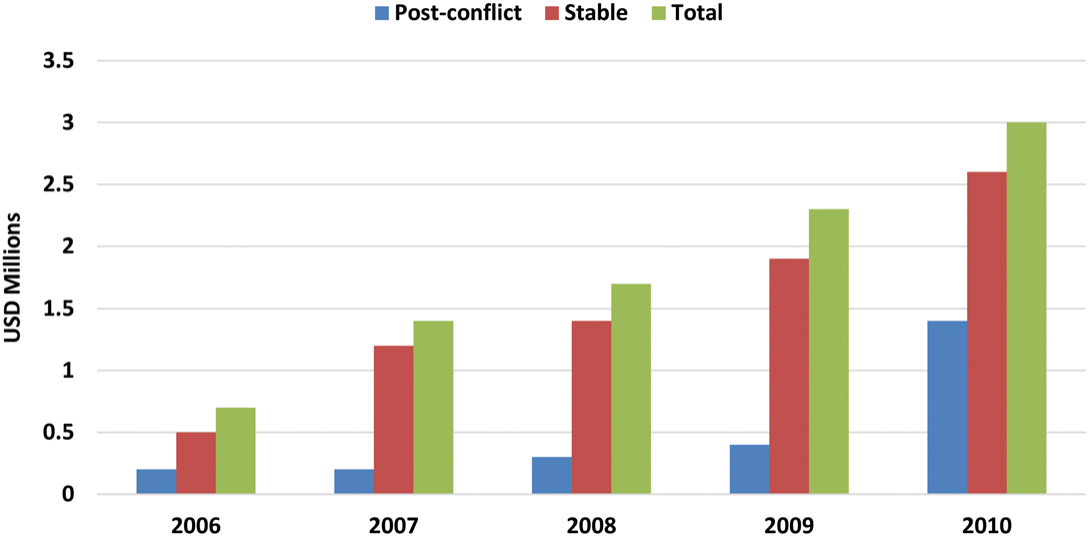
Contribution to national programs for onchocerciasis control (2006–2010).

The analysis of the national budgets shows the important contribution of APOC to onchocerciasis control, but simultaneously indicates that there is some way to go before governments sustain their own programs. The accumulating evidence suggests that in some countries there is a trend for increases in the level of government contributions (Fig 10). Unsurprisingly, this trend is clearest for the more stable counties with growing economies, and it seems likely that assistance will continue to be required by countries affected by conflict and other social shocks.

Fig 10 shows the national level of investment in onchocerciasis control over time, as reported by governments to the APOC Secretariat. The total amount shown is the aggregate for countries with stable economies and those countries emerging from conflict. The contribution is shown to increase in both types of countries but at a greater rate in those with stable and growing economies.

## Strengths and Challenges of the Trust Fund Mechanism

The APOC Trust Fund serves a useful role as a central funding mechanism for the various contributors and in particular for those who place a high premium on fiscal accountability. It presents a secure mechanism for obligating funds.

The fund benefits greatly from linking the comparative strengths of the World Bank and WHO as fiscal and health-implementing agencies, respectively. This strong partnership between WHO and the World Bank is one of the greatest strengths of the fund, but it contributes to what some donors consider a weakness since the inevitable layers of communication between the two entities present challenges for administration and reporting. This may not be an issue for donors whose primary concerns are that their funds are secure and are being used to implement closely supervised health programs, but it can be an issue for those donors seeking to track a direct connection between their contribution and a specific action at the country level.

Although all contributing parties (and participating governments) join into a standard agreement about the APOC Trust Fund and its management, not all development partners view the Fund in the same way. The history of the Fund shows that some partners use the Fund as one among many ways of supporting onchocerciasis control and elimination programs in Africa, while others use the Fund as their main and only conduit.

## Looking Forward

In January 2012, a high-level meeting in London, “Uniting to Combat Neglected Tropical Diseases,” announced a new public-private partnership to eliminate or control the seven preventable NTDs. The meeting, co-chaired by Bill Gates, from the Bill & Melinda Gates Foundation, and Sir Andrew Witty, CEO of GlaxoSmithKline, brought together 13 pharmaceutical companies as part of the continuing Gates-CEO Roundtable to enhance private sector investment in public health challenges in low-income countries. The meeting led to the “London Declaration on NTDs.” Among other pledges, the pharmaceutical companies collectively committed to donate most of the drugs necessary to control and eliminate all seven preventable NTDs in low-income countries.

At the 19th meeting of the JAF, held in Brazzaville in December 2013, and following a series of technical reviews requested by the participating countries over the previous two years, it was decided to establish a new integrated NTD regional program in 2016. This new regional initiative will actively seek to eliminate river blindness from most of Africa by 2025 and, by strengthening the same health system that successfully delivered treatment for river blindness, seek to co-implement treatments for the six other major preventable NTDs that can be addressed through mass drug administration. While the participating countries committed to increase national contributions to make this possible, they also asked for external help to create self-sustaining, community-based (including school-based) health systems that deal with the full range of preventable NTDs and other diseases of the poor that can be addressed through similar mechanisms. There was clear consensus among participating countries in favor of focusing first on onchocerciasis and lymphatic filariasis elimination with co-implementation for NTDs and health system strengthening [17,18]. This view was also supported by the 63rd Regional Committee of the WHO/AFRO. The institutional framework of this new initiative, provisionally named the Programme for the Elimination of Neglected Diseases in Africa (PENDA), will build upon the strength of APOC and is currently under discussion among partners.

In this new climate, there are indications that the APOC Trust Fund is finding new ways to contribute to onchocerciasis elimination and support the emerging NTD agenda (Box 2). There is a burgeoning movement led by philanthropists and civil society within Africa to contribute to Africa’s challenges. This interest is mirrored by government actions that recognize that disease is a shared problem and look beyond borders to support solutions. There are also some novel uses of the existing APOC Trust Fund to channel resources to a broader range of partners and to address NTD issues beyond onchocerciasis, such as supporting regional efforts to establish mapping and surveillance mechanisms.

**Box 2. New and Emerging Trends in the APOC Trust Fund**

**Nigerian philanthropy reaches out to the rest of Africa**

The Mission to Save the Helpless (MITOSATH) is a non-governmental development organization based in Jos, northern Nigeria, which has supported onchocerciasis control in Nigeria since 1996. At JAF 16 in Abuja in 2010, General Theophilus Danjuma, the Grand Patron and founder of MITOSATH, announced his intention to “extend the support of Nigerian philanthropy to control and eliminate river blindness throughout the continent of Africa.” He followed up with a US$1 million contribution to the Trust Fund and also challenged the government of Nigeria to follow his example. Building on the momentum of its private citizens and foundations, in December 2011, at a meeting of the APOC governing body (JAF) in Kuwait City, the government of Nigeria pledged US$5 million to the Trust Fund.

**International recognition for the work of APOC**

The global relevance of APOC’s work was recognized in 2011 with the prestigious Ant*ó*nio Champalimaud Vision Award from the Champalimaud Foundation in Lisbon, Portugal. This biennial award for the alleviation of visual problems in developing countries, is supported by the “Vision 2020: Right to Sight” initiative of the World Health Organization and the International Agency for the Prevention of Blindness. The Foundation President, Leonora Beleza, the former Portuguese Minister of Health, recognized APOC for its “outstanding contribution to the prevention, control, and fight against onchocerciasis…bringing health and hope to the poorest of the poor, especially those living in the remotest and underserved regions of Africa.” APOC and the Foundation decided to channel the €1 million through the Trust Fund to ensure equitable distribution to the beneficiaries of the APOC program.

**Strengthening the institutional reach**

In 2011, the Sabin Vaccine Institute, through a grant provided by the Global Network for Neglected Tropical Diseases, contributed US$1.2 million to the Trust Fund. In the words of Foundation President Ambassador Michael Marine, the aim is to “assist APOC’s efforts to include co-implementation of interventions against other preventable NTDs, and to facilitate institutional linkages around NTDs within the Africa region, especially with WHO/AFRO.” The subsequent success of this component has shown how the Trust Fund can be effective in supporting the implementation work of APOC within the broader regional leadership of WHO/AFRO.

**Broadening and extending the scope of APOC**

In 2012, the Trust Fund welcomed Sightsavers as its newest contributor. This international civil society organization, based in the UK and formerly known as the Royal Commonwealth Society for the Blind, has a long history with APOC as a member of the NGDO group. “We wanted to help APOC in its move to address other preventable NTDs, especially trachoma,” explained Sightsavers CEO Caroline Harper, “and decided the most effective way to do this was to step up to become for the next few years one of the contributors to the Trust Fund.” Sightsavers is the first of the donors to formally commit to supporting APOC beyond 2015.

Going forward, the key challenges for funding onchocerciasis control and elimination are to increase government commitments in the longer term, to convert this to government ownership, and to sustain resources in the interim while the countries gear up to address the challenges of onchocerciasis elimination and establish programs that address the NTDs more broadly [19,20]. A particular challenge will be identifying where the experience of APOC can contribute to integrating the many existing and planned NTD programs and activities.
